# Screening and Diagnosis Access for Neglected and Tropical Parasitic Diseases in Italy: A National Survey

**DOI:** 10.3390/tropicalmed10060153

**Published:** 2025-05-29

**Authors:** Agnese Comelli, Ester Oliva, Francesco Bernieri, Lorenzo Zammarchi, Libera Clemente, Luciana Petrullo, Guido Calleri, Fabrizio Bruschi, Annibale Raglio

**Affiliations:** 1 Italian Society of Tropical Medicine and Global Health, Steering Committee, 00161 Rome, Italy; agnese.comelli@gmail.com (A.C.); guido.calleri@aslcittaditorino.it (G.C.); 2 Microbiology Lab, AUSL IRCCS Santa Maria Nuova, 42123 Reggio Emilia, Italy; ester.oliva@ausl.re.it; 3 Comitato di Studio della Parassitologia Associazione Microbiologi Clinici Italiani, 20159 Milan, Italy; bernieri.fra@gmail.com (F.B.); liberaclemente@hotmail.com (L.C.); lucianapetrullo@yahoo.it (L.P.); annibaleraglio@gmail.com (A.R.); 4 Parasitology and Bacteriology Programs, Regional Reference Center for Quality Control of the Tuscany Region, 20145 Milan, Italy; 5 Infectious and Tropical Diseases Unit, Careggi University Hospital, 50134 Florence, Italy; 6 Department of Experimental and Clinical Medicine, University of Florence, 50134 Florence, Italy; 7 SSD Laboratorio Spoke Area Isontina, University Hospital Giuliano Isontina (ASUGI), 34074 Monfalcone, Italy; 8 UOS Parasitology, UOC Microbiology and Virology “D. Cotugno” Hospital of Azienda dei Colli of Naples, 80131 Naples, Italy; 9 Infectious and Tropical Diseases Unit, Amedeo Di Savoia Hospital, ASL Città di Torino, 10149 Turin, Italy; 10 Department of Translational Research and New Technologies in Medicine and Surgery, School of Medicine, Università di Pisa, 56126 Pisa, Italy; fabri.brus2@gmail.com; 11 Programma di Monitoraggio delle Parassitosi e Formulazione di Nuovi Algoritmi Diagnostici, AOU Pisana, 56126 Pisa, Italy

**Keywords:** migrant screening, neglected and tropical diseases, diagnostics access, parasitology

## Abstract

Background: The availability of laboratory tests to screen and diagnose migrants and travellers for neglected and tropical parasitic diseases significantly impacts individual and public health. Italian scientific societies for parasitology, tropical diseases, and global health developed a survey to assess number and geographical localisation of laboratories able to carry out adequate diagnostics. Methods: An open-ended and multiple-choice questionnaire was constructed and sent to 752 members working in Italian microbiology laboratories via scientific societies’ mailing lists. Data concerning malaria, cystic echinococcosis, leishmaniasis, schistosomiasis, strongyloidiasis, and Chagas disease were included. Results: Members from 96 laboratories replied. At least one laboratory responded from 18 out of 20 Italian regions. Serological tests for *Schistosoma* spp., *Strongyloides stercoralis*, *Trypanosoma cruzi*, *Echinococcus* spp., and *Leishmania* spp. are performed in <50% of responding laboratories. Only 56.6% of labs provide all three recommended tests for malaria diagnosis in the emergency room. Direct identification methods availability varies for Schistosoma eggs (75–95.8%), *S. stercoralis* larvae (53.1%), trypomastigotes (59.4%), and Leishmania amastigotes (53.1%). Geographical differences (mainly northern versus southern regions) were evident. Conclusions: The survey underlines the need to improve diagnosis for neglected and tropical diseases, to define a network of reference laboratories for testing less prevalent diseases, and to share information, education, and training for both clinicians and microbiologists/parasitologists.

## 1. Introduction

With the increase in world travel and access to varied populations and geographic areas, we continue to see more tropical diseases and infections outside areas of endemicity due to the rapidity with which people and organisms can move from one place to another. Travel has also become accessible and more affordable for many people worldwide, including those with compromised overall health status [1].

Many European countries are either the final destination or a transit point for migrants from low- and middle-income countries (LMICs). From an epidemiological perspective, this means that many migrants come from areas with different patterns of infectious diseases compared to the countries they are migrating to.

This observation underlines the importance of laboratory test availability to screen migrants for prevalent infectious diseases that significantly impact individual and public health, as recommended by several national and international guidelines [2,3].

According to a survey conducted in nine Italian sentinel centres, 4123 Neglected Tropical Diseases (NTDs) cases were diagnosed within a 7-year period (2011–2017), with schistosomiasis and strongyloidiasis being the most common NTDs, accounting for about one-third each of all the diagnosed cases, followed by Chagas disease caused by *Trypanosoma cruzi* [4].

Tilli et al. used hospital discharge records to retrieve the number of hospitalisations for some NTDs. Among 7195 hospitalisations, schistosomiasis, strongyloidiasis, and Chagas disease were the most common NTDs after leishmaniasis [5].

Besides migrants, those infections may also involve travellers and European residents who travel to visit friends and relatives (VFR). Malaria is the most diagnosed infection after travel to tropical countries, but schistosomiasis and, less frequently, Chagas and schistosomiasis may be imported infections into Europe—for example, the schistosomiasis outbreak in Corsica, France [6,7,8].

It is important to underline that some southern European countries, like Italy, share a similar disease endemicity with certain LMICs, including infectious diseases such as cystic echinococcosis (CE) [9,10,11] and leishmaniasis [12,13]. Furthermore, due to climate change, vector-borne diseases typical of tropical areas, like malaria or dengue, are at risk of emerging in these regions because of changing vector endemicity [14,15].

Indeed, most guidelines recommend specific population screening for the most prevalent tropical diseases, such as malaria, and some neglected tropical diseases (NTDs), such as schistosomiasis and strongyloidiasis.

In Table 1, a synthesis of screening recommendations is presented.

Screening is not the only activity that needs laboratory diagnostic tools. Diagnostic tests used in screening usually show good sensitivity but poorer specificity, which means that diagnostic confirmation with a second test may be necessary.

Moreover, alongside screening, malaria and NTDs may manifest in individuals displaying specific signs and symptoms. Specific diagnostic tools are needed to confirm the diagnosis and guide case management in this case. Outpatient and inpatient management may be necessary.

The current epidemiological scenario should lead to easier and broader access to diagnosis and treatment in Italy. In fact, diagnostic procedures in medical parasitology require a great interpretative experience with very few exceptions. Currently, very few procedures can be automated for routine laboratory use, and even when antigen, serological, or molecular biology tests are available, they do not always have sufficient sensitivity and specificity. Parasitological diagnosis can today be defined as multiparametric; the organism’s identification relies on morphologic characteristics that can be difficult to differentiate without experience. Serological tests and the recent development of bio-molecular tests can improve the microbiological diagnosis. Although morphology can be “learned” at the microscope, knowledge about the life cycle, epidemiology, infectivity, geographic range, clinical symptoms, range of illness, disease presentation depending on immune status, and recommended therapy is critical to the operation of any laboratory providing diagnostic services in medical parasitology.

Unfortunately, several issues hinder both treatment and laboratory diagnosis. The difficulties in accessing diagnosis outside of referral centres [21] are critical obstacles to following guidelines, providing patient care, testing to evaluate treatment response, and attempting to control the reintroduction of non-endemic diseases (such as malaria, strongyloidiasis, and schistosomiasis) or attempting to control and eliminate diseases like Chagas disease, malaria, and schistosomiasis.

From these premises, there is a need to teach and support human parasitological diagnostics [22]. The AMCLI Study Committee for Parasitology (CoSP) has provided comprehensive updates to intestinal, blood, and reticuloendothelial system parasitosis diagnostic pathways. The CoSP, in collaboration with the Italian Society of Tropical Medicine and Global Health (SIMET) and the Italian Society of Parasitology (SoIPa) contributed to mapping the availability of laboratory tests for the most prevalent and relevant neglected and tropical diseases in Italy.

This investigation aimed to create a network of national reference laboratories for parasitological diagnostics that could be contacted for consultation and/or specialist tests.

This work will focus on assessing access, types of tests used, and geographic distribution of diagnostic tools for malaria, schistosomiasis, strongyloidiasis, Chagas disease, CE, and leishmaniasis. Table 2 provides a literature review of Italian papers reporting the prevalence of the diseases focused on in this survey.

## 2. Materials and Methods

A questionnaire consisting of open-ended and multiple-choice questions was constructed using online Google Forms and was sent to the members of the three associations working in microbiology laboratories all over Italy via the AMCLI, SoIPa, and SIMET mailing lists (752 members). Not knowing the total number of laboratories in Italy, we decided to reach them through these three of the largest Italian associations that deal with parasitology.

The questionnaire is structured into 8 modules: laboratory details, pre-analytics, microscopic and cultural examinations for faecal parasites, microscopic and cultural examinations for blood parasites and parasites of the reticuloendothelial system and other body areas, antigen research, molecular biology, parasitological serology, and workload. Google Forms creates online Google Excel sheets that can then be downloaded by survey managers and modified for data analysis using the Microsoft Excel© 2021 application.

Overall, the questionnaire covered the diagnosis of faecal, blood, and reticuloendothelial system parasitosis. In this paper, we decided to summarise the survey’s results about malaria, schistosomiasis, strongyloidiasis, and Chagas disease because of their prevalence among the migrant population and CE and leishmaniasis because of an endemic transmission in Italy as well as among countries of origin of migrants.

The results were presented using frequencies, means, and medians.

## 3. Results

Ninety-six laboratories replied to the questionnaire. At least one laboratory responded from 18 out of 20 Italian regions. Sixteen (16.7%) were laboratories in the private sector, and eighty (83.3%) were in the public sector.

Eighty-one out of ninety-six laboratories (84.4%) belonged to hospitals.

**Figure 1:**
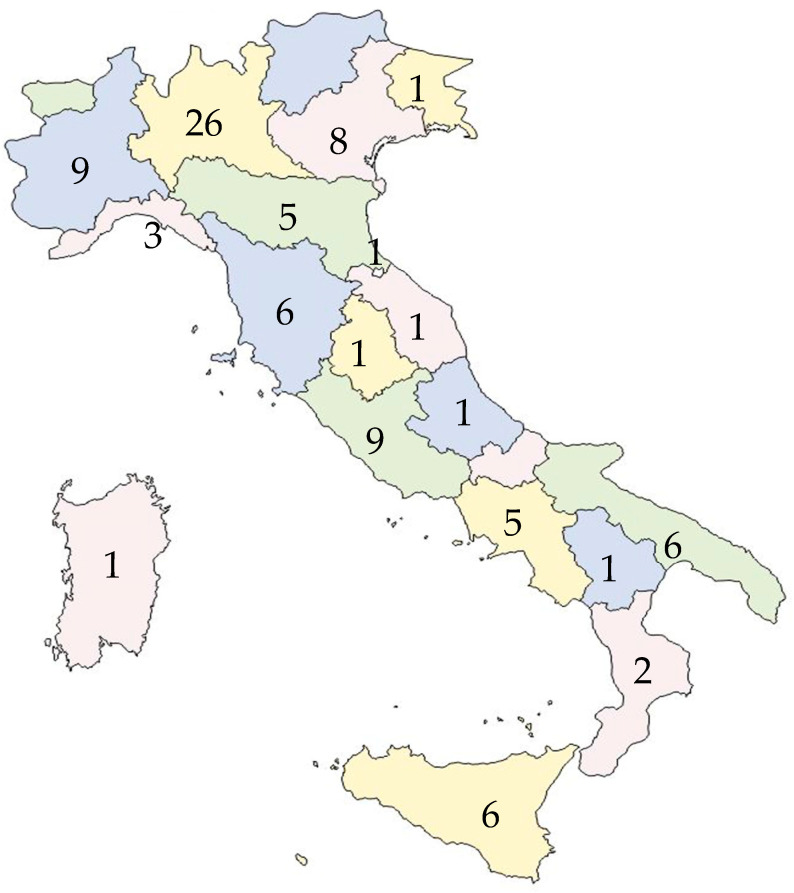
Number and the geographical distribution of the responding laboratories.

Figure 1 shows the number and the geographical distribution of the responding laboratories.

**Figure 2:**
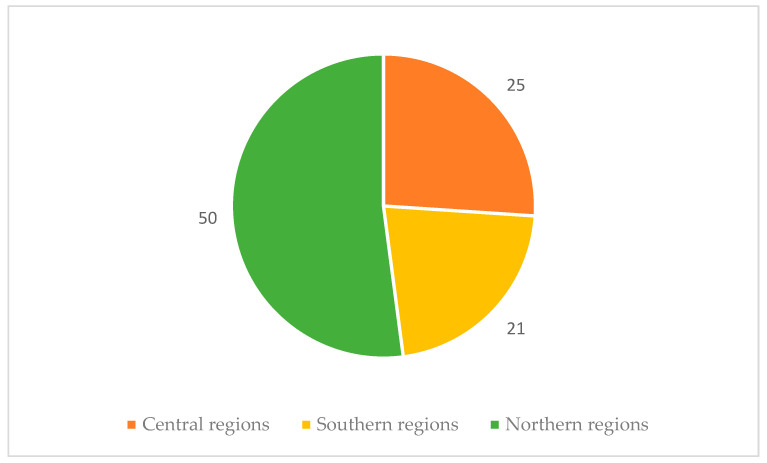
Distribution of participating laboratories according to geographical area.

Fifty out of ninety-six laboratories were in northern regions, with only 21 participating laboratories from southern regions (Figure 2).

Regarding the pre-analytical data, the questionnaire showed that only 36 out of 96 (37.5%) laboratories routinely receive the patient’s case history form that usually accompanies the clinical samples for parasitological investigations. When requesting parasitological tests, the attending physician should share clinical and epidemiological information. This helps the microbiologist choose the best testing techniques and request additional tests if needed.

### 3.1. Schistosomiasis

Seventy-five percent (72 out of 96) of laboratories can perform egg identification in urine. Among the respondents, 75% (72 out of 96) directly search for *S. haematobium* in urine, 87.5% of which carry out the centrifugation method. Ninety-two labs (95.8%) are able to look for eggs in stools. Twenty-seven laboratories (28.1%) had access to serology. For 27 laboratories, 10 different commercial kits are used: ELISA (8 laboratories), ICT (8 labs), WB (9 labs), IHA (3 labs), and IFA (1 lab). Two laboratories perform PCR on urine and 15 PCR on stools (four of them using a homemade assay).

### 3.2. Strongyloidiasis

Fifty-three out of ninety-six (53.1%) laboratories perform direct research for *S. stercoralis*, and the technique mainly used is agar plate culture examination (45.1%, 23 out of 51). Regarding the directly targeted research of this parasite, 23 laboratories perform only the culture, 10 perform the Berman test, 1 performs the Harada–Mori test. Ten laboratories out of ninety-six use two or more direct tests. Only 15 laboratories declare to use PCR. Among the respondents, 28 laboratories (29.2%) had access to serology. For these 28 laboratories, seven different commercial kits based on the ELISA method are used.

### 3.3. Chagas Disease

A total of 57 out of 96 laboratories (59.4%) perform direct research for *Trypanosoma* spp. The technique used is the research of trypomastigotes by microscopy with thin and thick blood smears. One laboratory (1.7%) uses the microhematocrit method. Serology for T. cruzi is available in 19 out of 96 labs (19.8%). In ten labs out of the nineteen that perform serology (52.6%), at least two different serologic tests are provided; in most cases, an ELISA test is associated with a second assay. In 3 labs out of 19 (15.8%), three different serological assays are available. In 6 out of 96 labs (6.3%), nucleic acid amplification tests (NAATs) for *T. cruzi* are available, and in 4 out of 6 cases, it is a homemade PCR.

### 3.4. Malaria

Malaria diagnosis is performed by 86% of the laboratories that answered the questionnaire. Most labs (66.3%) perform both thick and thin smears, and 31.3% only use the thin film. Among the laboratories that routinely perform the thick smear, 19 use the May–Grunwald Giemsa stain, which is not suitable for this type of preparation. The colouring diluted in water at pH 7.2 is instead used by 73.2% of the laboratories. A rapid ICT (immunochromatographic test) is widely available (71 laboratories, 73.9%), and a pan-malaria rapid test is used in all but two labs. Serology for *Plasmodium* spp. is available in 16 out of 96 labs (16.7%), with enzyme immunoassay assay being the most distributed (13/16, 81.2%). A total of 51 labs out of 83 (61.4%) are performing molecular biology for malaria diagnosis; 35 labs (68.6%) use only LAMP (loop-mediated isothermal amplification) method, 10 (19.6%) use both LAMP and PCR, and 5 (11.8%) use only PCR.

**Figure 3:**
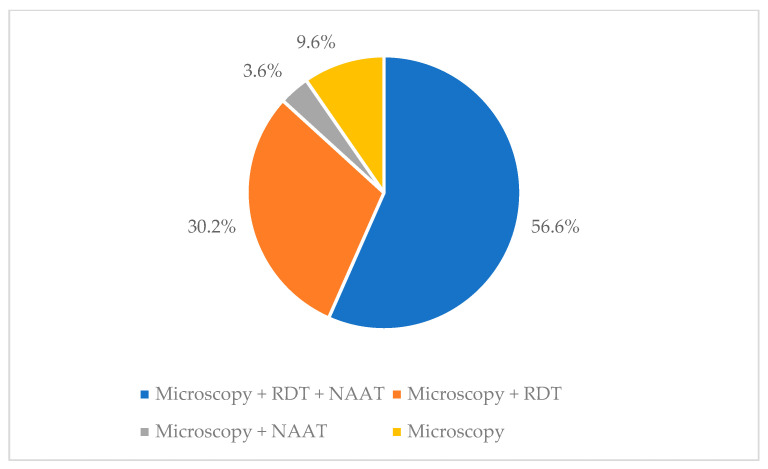
Different malaria diagnostic approaches among the national laboratories (footnote: RDT = Rapid Diagnostic Test, NAAT = Nucleic Acid Amplification Tests).

Figure 3 sums up the different diagnostic attitudes among the national laboratories.

### 3.5. Echinococcosis

Of the 96 laboratories that answered the survey, approximately half (47%) perform serology for this tapeworm, 14/96 perform the ELISA test, 11/96 use two or three combinations of tests to confirm the diagnosis (for example, 5 labs using ELISA tests in combination with the Western blot test). The direct search for parasites on cyst fluid is carried out by 46/96 laboratories, of which 30 declare that they only search for *Echinococcus* spp.

### 3.6. Leishmaniasis

Of 96 laboratories, 51 (53.1%) declare that they perform microscopic and/or culture research for *Leishmania* spp. in bone marrow, spleen, and lymph nodes. The research in 47 laboratories is performed by microscopic examination; only 4 perform both microscopy and culture. Microscopic and/or culture search for *Leishmania* spp. on the skin and subcutaneous material is performed by 37 laboratories; even in this case, only 4 laboratories perform both microscopy and culture. Serology is performed in 45 laboratories (47%); the most used technique is an ICT assay (9/45), followed by Western blot (6/45); 13 laboratories declare to use two or three tests to reach the diagnosis. PCR is performed in 24 labs (25%); 16/24 use commercially available kits, while the remaining 8 labs use in-house PCR.

### 3.7. Geographic Distribution of Tests

The distribution of testing for screening and diagnosing the six diseases shows significant inequality across the country.

**Figure 4:**
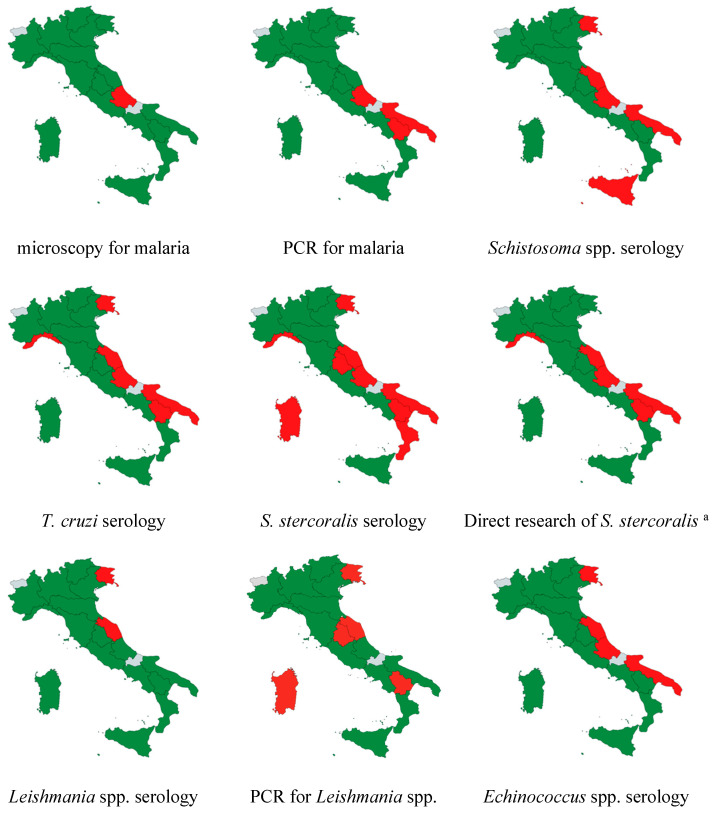
Regional availability of the different screening and diagnostic tests in the country. ^a^ culture, PCR, Baermann test, Harada–Mori test. Legend: Green = available at least in one responding lab, red = not available among the responding labs, grey = no laboratory in the region responded the survey.

**Figure 5:**
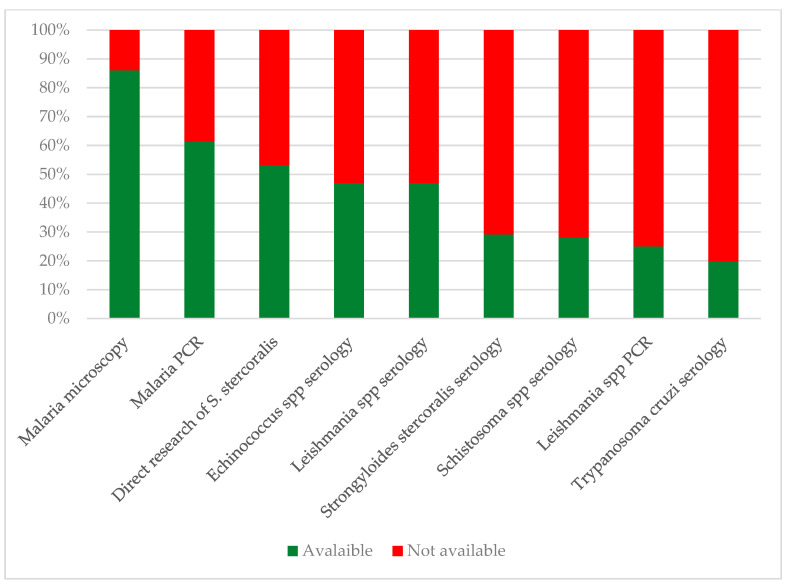
Availability of selected diagnostic tests for neglected and tropical parasitic diseases in Italian laboratories. Direct research of *S. stercoralis* includes at least one among culture, PCR, Baermann test, and Harada–Mori test.

Figure 4 and Figure 5 illustrate the nationwide availability of the tests throughout the country. Over 50% of laboratories offering screening or diagnostic tests are located in the north, approximately 30% are in central Italy, and less than 20% are in the south.

Only two laboratories conduct *Strongyloides* serology in the southern regions, specifically for four *Schistosoma* spp. and four *T. cruzi* serologies. Thirteen laboratories provide all three serologies (12 public hospitals and one private lab), with one hospital located in the southern regions.

## 4. Discussion

Advances in laboratory techniques have revolutionised parasite diagnostics over the past several decades. Although it is important to realise that not all laboratories can perform each procedure in the same way, it is very important to understand the pros and cons of the laboratory procedure and comply with the guidelines. For this reason, our purpose with the questionnaire was to map the availability of parasitological diagnostics tests to facilitate networking and consultation among different laboratories.

Geographical differences were evident either in the number of responding laboratories or in the availability of tests. In Italy, significant disparities in the quality of health services and economic inequalities are well-known [36]. However, we cannot conclude about a larger responding percentage from laboratories in the northern regions because the denominator is unknown. Indeed, the survey was sent to the registered emails of scientific society members rather than to each laboratory. This means that we cannot exclude the fact that a greater number of scientific society members work in the northern regions.

Widespread implementation of rapid antigen detection tests has greatly expanded access to tests for global parasitic threats such as malaria, even if there is still the need for a microscopic diagnosis, which is also useful for measuring parasitaemia.

Recently, the introduction of multiplex molecular panels for human gastrointestinal infections has enhanced the identification of common intestinal protozoa in faeces along with bacterial and viral pathogens. Despite the benefits provided by novel diagnostics in terms of sensitivity, automation, and early results, increased reliance on non-microscopy-based methods may contribute to the progressive, widespread loss of morphology expertise for morphological parasite identification. Such loss might negatively impact patient care, public health, and epidemiology. For example, for malaria diagnosis among the laboratories that routinely perform the thick smear, 19 out of 58 use the May–Grunwald Giemsa stain, which is not suitable for this type of preparation, and the colouring diluted in water at pH 7.2 is instead used by 73.2% of the laboratories.

Inadequate microscopy experience may lead to missed and inaccurate diagnoses and erroneous descriptions of emergent human parasitic diseases [37].

To guarantee correct and effective parasitological diagnostics, it is necessary to use the most reliable and targeted techniques, applied according to national and international guidelines [22]. The first step in complying with national and international guidelines on migrant screening is the availability of laboratory tests. Besides screening, neglected and tropical diseases, such as malaria, leishmaniasis, CE, schistosomiasis, strongyloidiasis, and Chagas disease, are prevalent in Italy, either among migrants, travellers, or current residents [4,5]. Although morphology can be “learned” at the microscope, knowledge about the life cycle, epidemiology, infectivity, geographic range, clinical symptoms, range of illness, disease presentation depending on immune status, and recommended therapy is critical to the operation of any laboratory providing diagnostic services in medical parasitology [1].

Unfortunately, we have observed a progressive decline in courses dedicated to parasitology in medical faculties all over Europe for a long time [38].

Despite the more common migrant routes arriving in the southern Italian regions as the first destination [39] and the recommendation of screening upon arrival [2,3], many laboratories in the south cannot offer the recommended serology assays.

In those areas, migrant screening for schistosomiasis, strongyloidiasis, and Chagas disease is a great challenge.

Moreover, there is a concern regarding the country’s approach to malaria diagnosis. Malaria is one of the few urgent infectious diseases to be diagnosed in the emergency room. Therefore, it is troubling that 14% of labs surveyed do not offer any tests for malaria diagnosis. Moreover, only 56.6% of labs performing at least one test for malaria may provide all three recommended tests (microscopy, rapid test, and NAAT) [22].

The results are not significantly different when considering other NTDs, such as leishmaniasis and CE. Even though these diseases are endemic in Italy, the availability of diagnostic tests is not consistent across the country. Serology for *Leishmania* spp. is carried out in less than 50% of labs, and only 4 out of 96 labs perform both culture and microscopy. Serology availability is geographically widespread; by contrast, PCR seems not to be available in regions such as Sardinia, which is among the top three regions in terms of the number of diagnoses. For echinococcosis, serology is performed by only half of the responding labs and seems to be lacking in Apulia, which ranks third in terms of reported cases.

Even though most Italian regions seem to perform most of the tests discussed in this paper, it does not mean those exams are easily accessible for patients.

Undocumented migrants in Italy face barriers in accessing tests. These individuals have the right to emergency and essential care through a dedicated healthcare code (STP), which must be obtained from the health authority. An exemption from direct healthcare costs applies in cases of extreme poverty.

The definition of essential care is not clearly defined. Although the prevention of chronic and debilitating diseases such as schistosomiasis, Chagas disease, and strongyloidiasis can be evidently listed as essential care, this lack of clarity could pose a barrier to migrant screening.

In Italy, the Essential Levels of Assistance (LEA) represent the list of lab tests the public health care system must make available to people. In 2017, with a dedicated decree, this list was updated to include *Schistosoma* spp., *Strongyloides* spp., and Chagas disease serology [40]. Unfortunately, after seven years, this decree has not been applied at the national level. However, only some regions have already implemented the test, relying on their own fund. This means that in most regions, those tests can be easily prescribed during hospitalisation but not in the outpatient setting or primary health care services, which are the preferred sites for screening.

For Chagas disease, for instance, the Italian law requests the blood donor centres to perform serology tests since 2015 [16,41]. By contrast, our survey showed that most (80.2%) of the responding microbiology laboratories in this survey do not perform Chagas serology.

A national survey published by the Italian National Blood Centre in 2022 involving 81% of Italian blood donor centres showed that blood donor screening seems to be well implemented at national level [42].

The survey showed that 98% of the responding centres performing collection/validation of blood and blood components declared to be able to perform *T. cruzi* serology for blood donors at risk. Studies that analysed seroprevalence among blood donors reported a positive rate from 0.06 to 0.5% [33,42]. However, the access to serology for diagnostic purposes (i.e., migrants from endemic areas with cardiological or gastrointestinal symptoms) or screening of different populations (i.e., pregnant women) is completely different since a microbiology laboratory should test samples from these subjects while it is not possible to test these samples in a blood donor centre.

For this reason, it can happen that in the same Italian city, an Italian blood donor who returned from a two-week trekking trip in Peru will be subjected to mandatory testing for *T. cruzi* antibody before blood donation, while it may be challenging to test an asymptomatic Bolivian pregnant woman or a Salvadorian man with arrhythmic disturbance.

An established network of laboratories performing different tests for different diseases may be a solution to overcome this lack of equity and avoid wasting resources on providing diagnostic kits in contexts where the number of requests is expected to be low according to the origin of migrants in the area covered by the laboratory. To ensure the interruption of transmission in non-endemic countries, autochthonous recipients of blood or organs must be protected from transmission, as well as those born in endemic countries or with siblings coming from those geographical areas. There is an urgent need to organise a network of general practitioners, obstetricians, paediatricians, infectious disease experts, and laboratories to ensure effective diagnostics and clinical protocols, preventing an overload of unused kits in inexperienced centres.

To complicate the picture, even when the diagnosis is made, early and appropriate treatment for the above-mentioned diseases is hindered by the lack of access to drugs, most of which are included in the WHO’s essential drug list [43,44].

Our study has some limitations. We cannot precisely estimate the percentage of responding laboratories because the denominator is unknown. Indeed, the survey was sent to the registered email of scientific society members and not to each laboratory. This affects the generalisability of the results even though the significant difference in test availability between northern and southern regions seems to support the likelihood of this finding. Another negative aspect is that we do not know the total number of microbiology laboratories, either public or private, and their distribution in Italy. Finally, we did not explicitly ask the laboratory if they are able to send samples to other laboratories to perform specific tests not available locally. Indeed, it could be another way for people to access good-quality tests. However, in the available free text in the survey sheet, only one laboratory stated that samples could be sent to the Italian National Institute for some tests and, in the survey, the laboratory was considered as an accessible testing point.

## 5. Conclusions

The results of this survey should support an analysis of the barriers and facilitators that explain this geographic difference. Even though the overall results may merely represent an example of the well-known healthcare access disparities among Italian regions [36], these exacerbate the inequalities in access for the migrant population.

We can pinpoint several unmet needs, as follows: practical education regarding parasite diagnoses; conducting audits and external assessments to ensure that at least the most critical diagnoses, like malaria, are performed; establishing reference laboratories at a regional level for centralising specific and non-urgent tests; and creating a network for sharing information, education, and training for both clinicians and microbiologists/parasitologists.

These activities must be undertaken to enhance diagnosis and treatment, ensuring compliance with international and national recommendations.

## Figures and Tables

**Table 1:** Synthesis of screening recommendation for migrants (national and European guidelines): schistosomiasis, strongyloidiasis, malaria, Chagas disease, and leishmaniasis.

**Schistosomiasis**
Post-arrival serological screening for migrants coming from endemic areas [2,3] Serological screening of Solid Organ Donors coming from endemic countries [16]
**Strongyloidiasis**
Post-arrival serologic screening for migrants from endemic areas [2,3] Combined screening (serology PLUS stool culture OR Polymerase Chain Reaction on stool) of individuals born or who have lived in endemic areas, including Italian elderly people, who undergo immunosuppression [17]. Universal serological screening of Solid Organ Donors [16]
**Malaria**
Active detection for signs and symptoms of clinical malaria in migrants upon arrival [3] Screening by RDT or PCR and microscopy in subjects who were born or who have lived or travelled in endemic areas who present aspecific signs such as fever, thrombocytopenia, and/or splenomegaly [3] Serological screening for blood donors who have lived for 6 months or more in an endemic area during their life or who have suffered from malaria [18] Combined serological and NAAT screening of Solid Organ Donors from endemic countries [16]
**Chagas disease**
Serological screening of blood donors born in an endemic country (or whose mother was born in an endemic country) and subjects having travelled to an endemic country who have engaged in at risk activities and subjects who have received a blood transfusion in an endemic country [16] Serological screening of Solid Organ Donors borne in an endemic country (or whose mother was borne in an endemic country) or subjects who have lived for a prolonged period in an endemic country [16] Serological screening of pregnant women borne in an endemic country (or whose mother was borne in an endemic country), pregnant women who have received a blood transfusion in an endemic country and pregnant women who have resided for at least 6 months in an endemic country [19]. In order to eliminate congenital transmission of *T. cruzi*, efforts should be focused on five population groups living within or outside of Latin America (as defined previously), namely the following: girls and female adolescents (pre–conceptional phase), women of fertile age not yet pregnant (childbearing age), pregnant women, neonates/infants born to infected mothers, and relatives and other children born to infected mothers (siblings) [20]
**Leishmaniasis**
Combined serological and NAT screening of Solid Organ Donors from endemic countries [16]

**Table 2:** Review of prevalence and incidence data in Italy.

Schistosomiasis	In Italy, schistosomiasis is highly prevalent among migrants coming from endemic countries. Zammarchi et al. reported that 1433 out of 4123 NTD cases diagnosed in nine Italian sentinel centres over a seven-year period were due to schistosomiasis [4].Reported seroprevalence ranges from 5.9% to 34% [23,24,25,26,27], with higher prevalence among migrants coming from Mali (63.6% to 72.1%) [25,27] and Ivory Coast (43.1% to 48%) [25,27].
Strongyloidiasis	Strongyloidiasis can affect migrants from a wide geographical area, as well as the elderly from former endemic countries like Italy. Zammarchi et al. reported that 1282 out of 4123 NTD cases diagnosed in nine Italian sentinel centres over a seven-year period were due to strongyloidiasis [4]. Reported seroprevalence ranges from 2.7% to 19.9% [23,26,28,29]. In a study assessing serology among people accidentally presenting with eosinophilia, 8% of Italians (born before 1952) were seropositive [28].
Chagas disease	Zammarchi et al. reported that 581 out of 4123 NTD cases diagnosed in nine Italian sentinel centres over a seven-year period were due to Chagas disease [4].Reported mean seroprevalence ranges from 0.5% among blood donors to 3.9%–9.6% among migrants [23,30,31,32,33]. Seroprevalence among people originating from Bolivia is more than double (23.5% to 30.3%) [30,31,32]
Malaria	The last data regarding the number of malaria diagnoses in Italy date back to 2022 [34,35]. Total cases per year ranged from 701 to 888 malaria cases, mainly due to *P. falciparum*, in the 2013–2017 years report [34]. More than 80% of cases involved non-Italian patients. Most reported cases originated from Northern Italy; however, the comparison between official notifications and hospital discharge records revealed an underreporting from southern and central Italy centres. This highlights the prevalence of imported malaria cases in those regions as well [34]. In line with the reduced volume of international travel during the SARS-CoV-2 pandemic, the cases reported in Italy fell to 181 in 2020 and rapidly increased in the following years with the reopening of movement (571 cases reported in 2022) [35].
Echinococcosis	Since establishing the Italian Registry of Cystic Echinococcosis (RIEC), a more precise estimate of echinococcosis cases has been possible. A first publication in 2015 reported 346 patients enrolled in 11 centres between 2012 and 2014 [11]. A recent systematic review found a total of 15,489 cases of CE between 1997 and 2021 in Italy. As previously mentioned regarding malaria, there is also evident under-notification for CE when compared to HDRs (15,489 cases reported versus 24,651 CE HDRs) [10]. Most cases are diagnosed in patients from southern regions [9,10,11]. A paper detailing the regional distribution from a participating centre in northern Italy revealed that over 70% of native Italian patients were born in southern regions, with the top three being Sicily, Calabria, and Apulia [11]
Leishmaniasis	According to the ECDC surveillance system, Italy belongs to European countries with moderate-to-high endemicity for leishmaniases [13]. Overall, 1059 visceral leishmaniasis (VL) cases and 624 cutaneous leishmaniasis (CL) cases were recorded from 2005 to 2020 (autochthonous plus imported cases) [13].When looking at the regional distribution of cases by the HDRs, the highest incidences are from Sicily, Liguria, and Sardinia [13].

## Data Availability

The original contributions presented in this study are included in the article. Further inquiries can be directed to the corresponding author.
